# Genetic association of leukocyte telomere length with Graves’ disease in Biobank Japan: A two-sample Mendelian randomization study

**DOI:** 10.3389/fimmu.2022.998102

**Published:** 2022-09-29

**Authors:** Meijie Ye, Yu Wang, Yiqiang Zhan

**Affiliations:** Department of Epidemiology, School of Public Health (Shenzhen), Sun Yat-Sen University, Shenzhen, China

**Keywords:** Graves’ disease, leukocyte telomere length, Mendelian randomization analysis, single nucleotide polymorphism, risk factor

## Abstract

**Background:**

Telomere length (TL) has been recognized to be fundamental to the risk of autoimmune disorders. However, the role of leukocyte TL in Graves’ disease has not yet been fully elucidated. In the study, we exploited the two-sample Mendelian randomization (MR) design to evaluate the causal effect of leukocyte TL on the risk of Graves’ disease.

**Methods:**

Genome-wide association study (GWAS) data of leukocyte TL from the Singapore Chinese Health Study (SCHS) cohort and Graves’ disease from Biobank Japan (BBJ, 2176 cases and 210,277 controls) were analyzed. Nine single nucleotide polymorphisms (SNPs) were selected as instrumental variables (IVs) for TL. We used the inverse variance weighted (IVW) approach as the main estimator and MR-Egger regression, weighted median, simple mode, and weighed mode methods as complementary estimators. Horizontal pleiotropy was assessed using the intercept from MR-Egger.

**Results:**

The analysis demonstrated that genetically predicted longer leukocyte TL was causally associated with a lower risk of Graves’ disease using the IVW method (odds ratio [OR]: 1.64, 95% confidence interval [CI]: 1.23-2.17, *P*=2.27e-04, and other complementary MR approaches achieved similar results. The intercept from the MR-Egger analysis provided no noticeable evidence of horizontal pleiotropy (*β*=0.02, *P*=0.641). MR-PRESSO method reported no outliers (*P*=0.266).

**Conclusions:**

Our results provided evidence to support a genetic predisposition to shorter leukocyte TL with an increased risk of Graves’ disease. Further studies are warranted to explore the mechanism underlying the association.

## Introduction

Graves’ disease is the most frequent cause of persistent hyperthyroidism due to the overproduction of thyroid hormones (1–3). Graves’ disease is an organ-specific autoimmune thyroid disorder that is characterized by breaking immune tolerance against thyroid antigens resulting in thyroid autoimmunity (4). The primary clinical symptoms or signs of Graves’ disease are diffuse thyroid enlargement and overaction, ophthalmopathy, pretibial myxedema, palpitations, weight loss, and others (5). The incidence of Graves’ disease is 20-40 cases per 100,000 person-years worldwide (6), and is more common in women than men, which approximately affects 0.2% of men and 2% of women (7, 8). Graves’ disease is commonly observed in women aged 20-50 years, whilst all ages could be affected (6). Although the autoimmune mechanism of Graves’ disease has been proposed for several decades, its etiology has not been well accepted and needs to be further elucidated.

Telomeres, the terminal nucleoprotein-DNA complexes of eukaryotic chromosomes, play crucial and pervasive roles in capping and protecting chromosome DNA ends (9, 10). Telomeres, to a certain extent, can maintain the integrity of the replicative capacity of the chromosomes after each cell cycle (11). Telomeres progressively shorten with each cellular division cycle until eventually reaching a critically short length or sufficiently damaged, which triggers subsequently cellular senescence or apoptosis (12). Consequently, telomere length (TL) has been proposed as an underlying biomarker for cellular aging and mortality (13–15). Furthermore, telomere attrition might give rise to potential cell changes, block cellular division, and interfere with the normal function of tissues (12). Phenome-wide analyses provided evidence for association of shorter leukocyte TL with increased risk of thyroid disorders in more than 350,000 UK participants (16). However, whether there is a potential causal effect on leukocyte TL and Graves’ disease has not yet been investigated.

**Figure 1:**
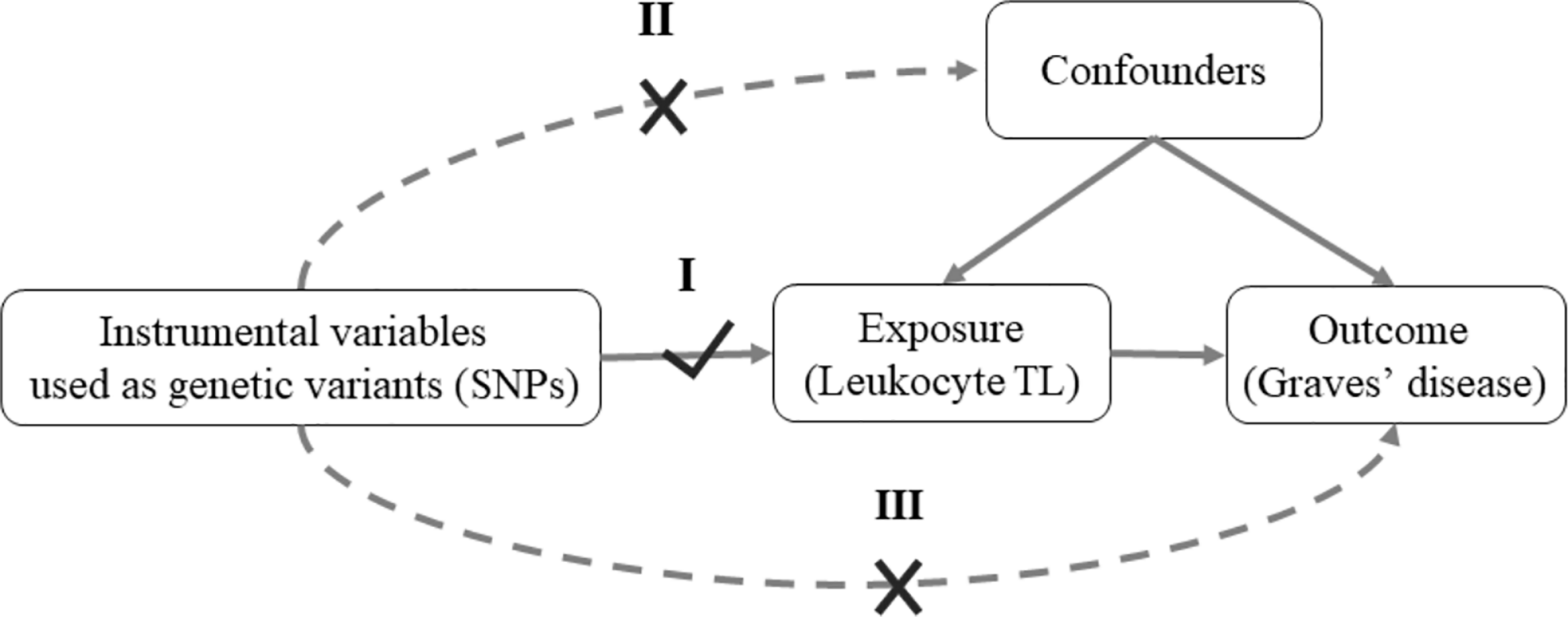
Mendelian randomization model of leukocyte TL and risk of Graves’ disease. MR depends on three assumptions: I. Relevance: The genetic variants are associated with the exposure of interest (Leukocyte TL); II. Independence: The genetic variants are not associated with confounders; III. Exclusion restriction: The genetic variants affect outcome of interest (Graves’ disease) except through their potential effects on the exposure (Leukocyte TL). Solid arrows represent causal effects; dashed arrows represent causal effects that are particularly violated by the IV assumptions. SNPs, single-nucleotide polymorphisms; TL, telomere length.

Mendelian randomization (MR) study, an epidemiological method, could minimize potential bias due to confounding and reverse causation (17). MR provided a novel opportunity to explore the potential causal association between an exposure of interest and outcome in an observational study (18). It relies on three core assumptions (**Figure 1**): (I) the instrumental variables are associated with the exposure of interest (e.g. leukocyte TL); (II) the instrumental variables are not associated with confounders; (III) the effects of instrumental variables on the outcome (e.g. Graves’ disease) are only through the exposure of interest. To the best of our knowledge, there is no study examining leukocyte TL and Graves’ disease using the MR approach.

In the present study, we identified nine SNPs to be of genome-wide significance for leukocyte TL in a large-scale genome-wide association study (GWAS) from the Singapore Chinese Health Study (SCHS) cohort and examined its association with Graves’ disease in Biobank Japan.

## Materials and methods

### Instrumental variable selection for leukocyte TL

The Singapore Chinese Health Study is a large prospective cohort and recruited 63,257 Singaporean Chinese men and women aged 45-75 years from April 1993 to December 1998. TL was assessed in a subset of 23,096 individuals of Chinese ancestry and a GWAS was conducted (19, 20). Multiplex quantitative PCR (qPCR) was utilized to measure relative telomere length (21). For single-nucleotide polymorphism (SNPs) quality control, sex-linked and mitochondrial SNPs were omitted, leaving autosomal SNPs in this study. In total, the genetic assessment identified ten independent SNPs (*P*<5e-08) to be of genome-wide significance with TL, and the annotated genes play crucial roles in the biological mechanisms of the telomere.

### GWAS summary statistics for Graves’ disease

Genetic association data on Graves’ disease was obtained from a large-scale GWAS in a Japanese population, including 2,176 cases (in 597 male patients and 1,579 female patients) who participated in the BioBank Japan Project (BBJ) and 210,277 population-based controls of Asian ancestry (22). BBJ, one of the largest non-European biobanks, collects collaboratively DNA and serum samples from 12 Japanese medical institutions and comprises a large cohort of approximately 200,000 participants (23, 24).

For quality control of samples, samples with a call rate <0.98 were excluded. Subsequently, for quality control of genotypes, variants that were call rate <99%, with Hardy-Weinberg equilibrium (HWE) *P <*1.0×10^-6^, and less than five heterozygotes were excluded (22). Among identified nine independent SNPs with leukocyte TL in Singaporean Chinese individuals, the associations of SNPs with TL and Graves’ disease were harmonized to make sure that the direction of effects reflected the same alleles. These GWAS data for Graves’ disease were publicly available online (http://jenger.riken.jp/65/).

### Statistical analysis

MR analysis uses genetic variants (SNPs) as instrumental variables to assess the causal effect of exposure of interest on the outcome. A two-sample MR design was performed in the present study. We combined the GWAS summary statistics (*β* coefficients and standard errors) to estimate the causal association between leukocyte TL and Graves’ disease using different MR approaches. The inverse variance weighted (IVW) analysis was selected as the primary method to evaluate the causal association. The IVW estimate for combining the ratio estimate on multiple genetic variants is the weighted average of ratio estimates of the genetically causal association of outcome with exposure for each SNP (25). Other MR approaches take into account different types of genetic pleiotropy and are based on potentially different assumptions, which were conducted to examine the robustness of the results. For example, a weighted median estimator (WME) combines multiple genetic variants into a single causal estimate. The weighted median estimator may be able to provide consistent and robust estimates, although up to 50% of the weights are from invalid instrumental variables (26).

Further, the MR-Egger regression can correct bias in the presence of directional pleiotropy and heterogeneity and provide a less biased estimate for effect estimates (27). The MR-Egger regression provides a valid test of directional pleiotropy, along with a robust analysis of invalid instruments (28). When the SNPs have noticeably non-pleiotropic associations with the outcome, the coefficient of the slope from MR-Egger regression might strengthen the confidence of the causal effect (29). However, the MR-Egger method is more sensitive to the effects of SNPs on exposure and outcome, which is of lower statistical power with a wider confidence interval compared to the IVW analysis. In addition, we applied methods of visualization to evaluate causal estimates. The scatter plot visually depicts causal effects on exposure of interest and outcome *via* selected MR approaches. Besides, the forest plots display the causal estimate of a single SNP, which allow for a visual inspection of heterogeneity around the overall causal estimate from all SNPs (30).

The study was performed according to the Strengthening the Reporting of Observational Studies in Epidemiology Using Mendelian Randomization (STROBE-MR) Statement (31).

Sensitivity analyses primarily are comprised of tests for heterogeneity, genetic pleiotropy, and the leave-one-out analysis. Firstly, heterogeneity in causal effects among IVs is an indicator of potential violations of MR assumptions, which can be calculated for the IVW and MR-Egger estimates to assess horizontal pleiotropy (32). The *p*-value of Cochran’s Q statistic was used to test for heterogeneity, whilst the *I^2^* statistic assesses the magnitude of heterogeneity. Secondly, for the estimate of genetic pleiotropy, the intercept of MR-Egger regression was used to examine horizontal pleiotropy. Besides, the MR pleiotropy residual sum and outlier (MR-PRESSO) test is used to detect and correct for outliers of horizontal pleiotropy in MR analysis (33). Thirdly, the effect estimates on exposure and outcome for SNPs are visually plotted to test probable outlier genetic variants. The leave-one-out analysis is performed by removing each SNP and the remaining SNPs were further tested. The fluctuation of the estimates after removing each SNP implies the probability of identifying any outliers. The funnel plot is commonly used in the meta-analysis, in which an estimate for a single SNP is plotted against its precision (34). The funnel plot can be applied to conduct a visual inspection for asymmetry, which may be an indication of violations of the MR assumption *via* horizontal pleiotropy (27).

We conducted power calculation for MR analysis *via* using an online power calculator (https://shiny.cnsgenomics.com/mRnd/) and found that the statistical power for the present study was 91% by assuming the odds ratio for Graves’ disease was 1.5 per SD decrease in TL. All statistical analyses were conducted in R version 4.1.1. The use of the *TwoSampleMR* package tested the MR approaches. The *P*-value <0.05 was significantly statistical.

### Ethical approval and informed consent

Our study took advantage of publicly available GWAS summary data. Informed consent was obtained from all participants, and each study was approved by their institutional ethics committees. The original data was not collected for this manuscript, and thus, no ethical approval was required.

## Results

### Selection of instrumental variables

Ten independent SNPs with leukocyte TL association were originally identified, then the associations of SNPs with exposure and outcome were harmonized to make sure whether there were the same effect or risk alleles. However, one SNP (rs28365964 and its proxy SNPs) was missing in the Graves’ disease GWAS, and it was therefore excluded. Eventually, the remaining nine SNPs were available for instrumental variables and their effects on exposure and outcome were extracted and harmonized. The summary statistics of nine genetic variants was shown in **Table 1**.

**Table 1:** SNPs used as instrument variables and its association of leukocyte TL with Grave’s disease.

SNP	Chromosome	Position	Risk allele	Other allele	SNP- leukocyte TL		SNP- Grave’s disease	
					*β*	SE (*β*)	*β*	SE (*β*)
rs3219104	1	226562621	A	C	0.074	0.009	-0.087	0.031
rs2293607	3	169482335	T	C	-0.120	0.009	0.073	0.032
rs10857352	4	164101482	A	G	0.058	0.010	0.036	0.039
rs7705526	5	1285974	C	A	0.118	0.009	-0.063	0.036
rs7776744	7	124599749	A	G	-0.058	0.009	0.017	0.032
rs12415148	10	105680586	T	C	0.204	0.020	-0.064	0.061
rs227080	11	108247888	A	G	-0.060	0.009	0.056	0.031
rs41293836	14	24721327	C	T	0.233	0.017	-0.139	0.063
rs41309367	20	62309554	C	T	-0.058	0.010	-0.051	0.037

SNP, single nucleotide polymorphism; TL, telomere length.

### The causal effect of leukocyte TL on Graves’ disease

**Figure 2:**
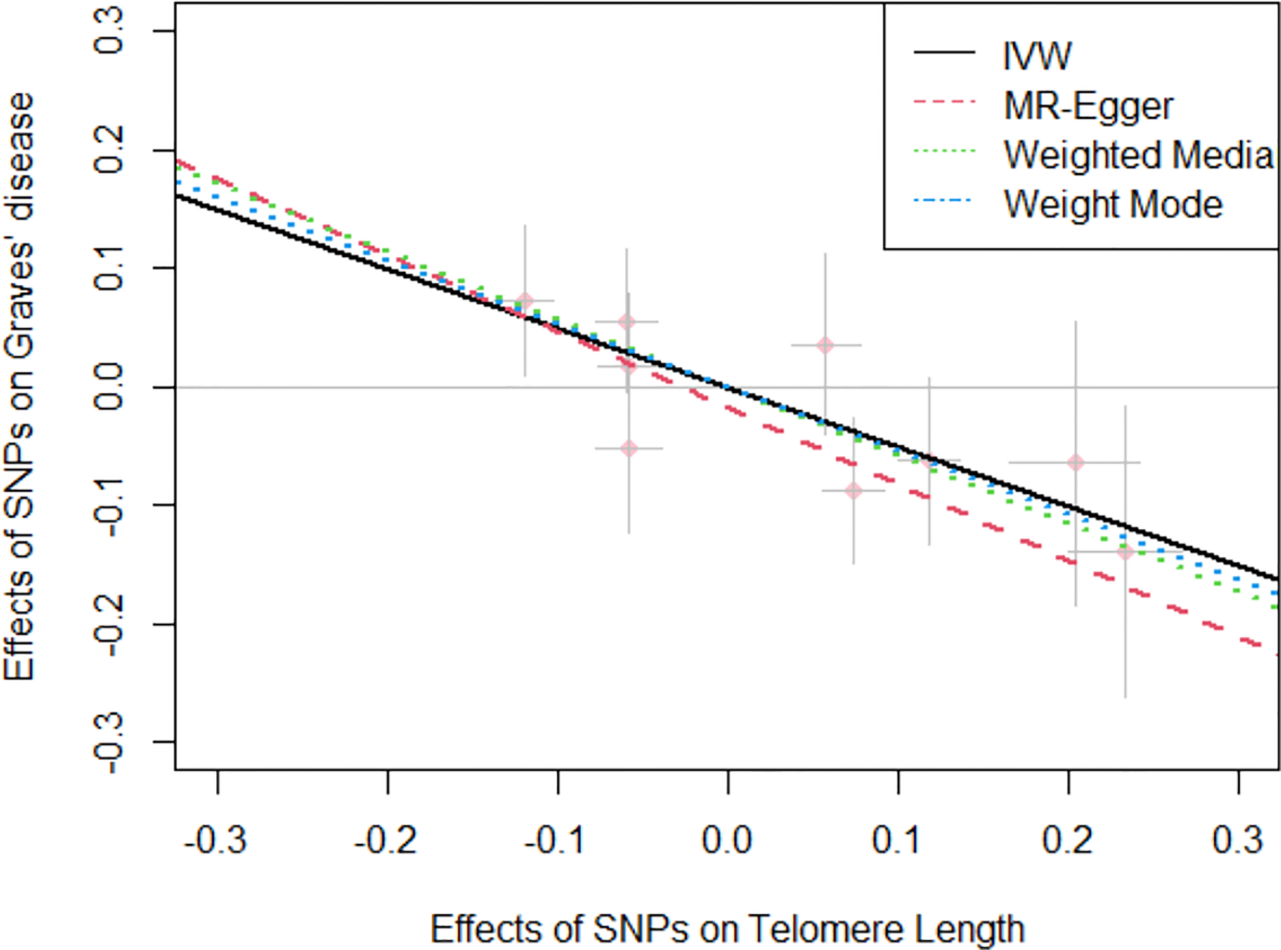
Scatter plot for the negative effects of SNPs on telomere length and Graves’ disease. The horizontal and vertical axes represent the effects of each genetic variant on both telomere length and Graves’ disease. The grey lines around the solid pink points denote the corresponding 95% CI for the effects. The slopes of solid lines show the effect estimates from four MR approaches. IVW, inverse variance weighted; MR, Mendelian randomization; SNPs, single nucleotide polymorphisms; CI, confidence interval.

Overall, in the primary analysis using IVW that combines the ratio estimate on multiple genetic variants, genetically predicted shorter leukocyte TL was causally associated with a higher risk of Graves’ disease (OR: 1.64, 95% CI: 1.23-2.17, *P*=2.27e-04). The odds ratio of Graves’ disease per 1-standard deviation decrease in genetically predicted leukocyte TL was 1.64 (**Table 2**). Results obtained using weighted median (OR:1.79, 95% CI: 1.30-2.44, *P*=6.16e-04), simple mode (OR:1.64, 95% CI:1.10-2.50, *P*=0.043), and weighted mode (OR:1.73, 95% CI: 1.23-2.38, *P*=0.013) approaches were in the same direction, with comparable point estimates and confidence intervals. These causal estimates were further displayed in a scatter plot (**Figure 2**).

**Table 2:** Associations between leukocyte TL and Graves’ disease using Mendelian randomization method.

Method	beta	se	*OR* (95%CI)	*P*
Inverse variance weighted (IVW)	0.50	0.15	1.64 (1.23-2.17)	2.27e-04
Weighted median	0.58	0.16	1.79 (1.30-2.44)	6.16e-04
Simple mode	0.50	0.21	1.64 (1.10-2.50)	0.043
Weighted mode	0.54	0.17	1.73 (1.23-2.38)	0.013
MR Egger_slope_	0.65	0.34	1.92 (0.99-3.70)	0.097
MR Egger_intercept_	0.02	0.03	NA	0.641

IVW, inverse variance weighted; MR, Mendelian randomization; PRESSO, pleiotropy residual sum and outlier; OR, odds ratio; CI, confidence interval; NA, not applicable.

### Pleiotropy and sensitivity analysis

**Figure 3:**
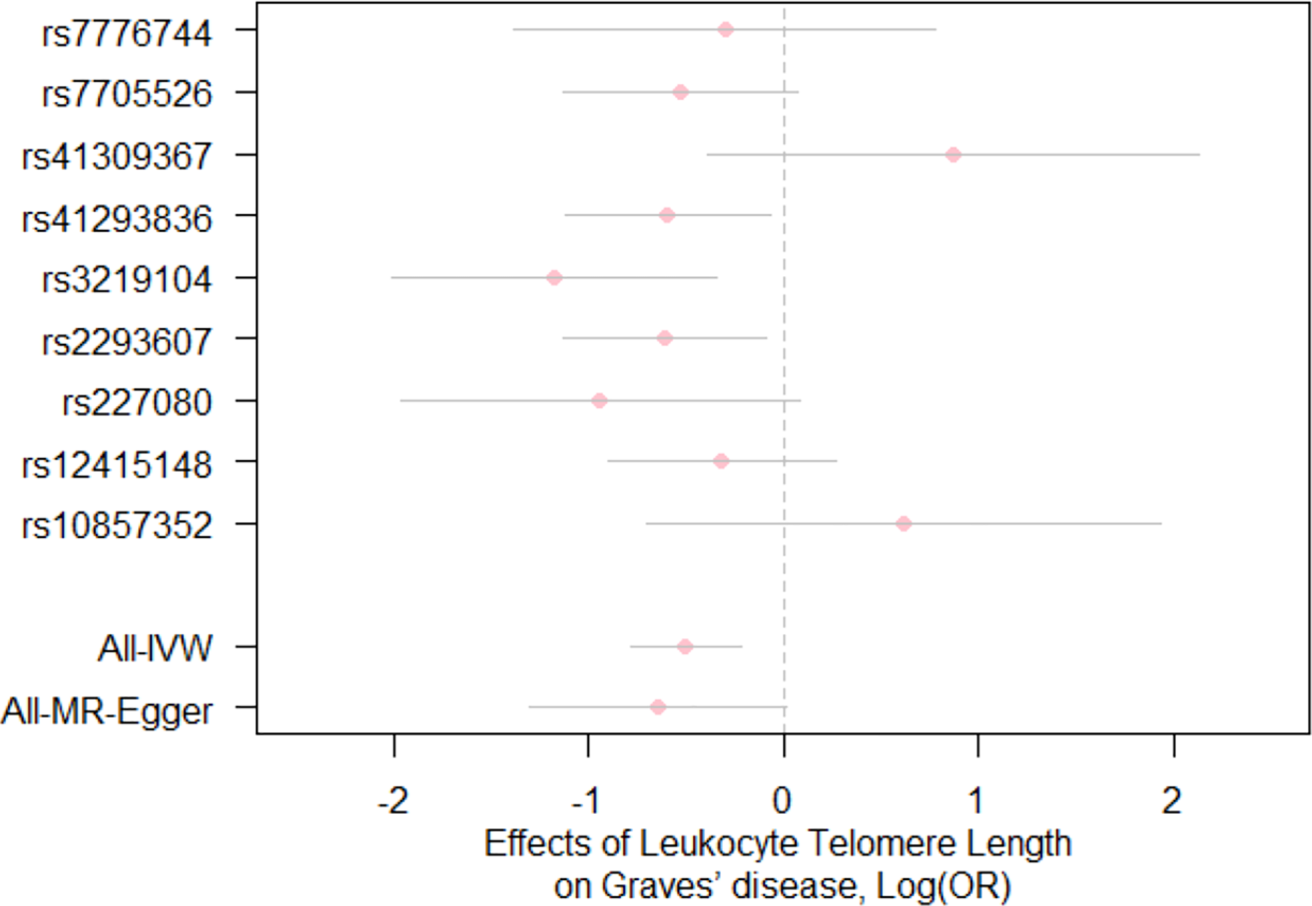
Forest plot for the effects of SNPs on telomere length and Graves’ disease. Each pink dot and grey bar represent the effect estimate and 95% CI for each SNP, respectively. The overall effect estimates and 95% CI are indicated by using the IVW and MR-Egger approaches. IVW, inverse variance weighted; MR, Mendelian randomization; OR, odds ratio; SNPs, single nucleotide polymorphisms; CI, confidence interval.

**Figure 4:**
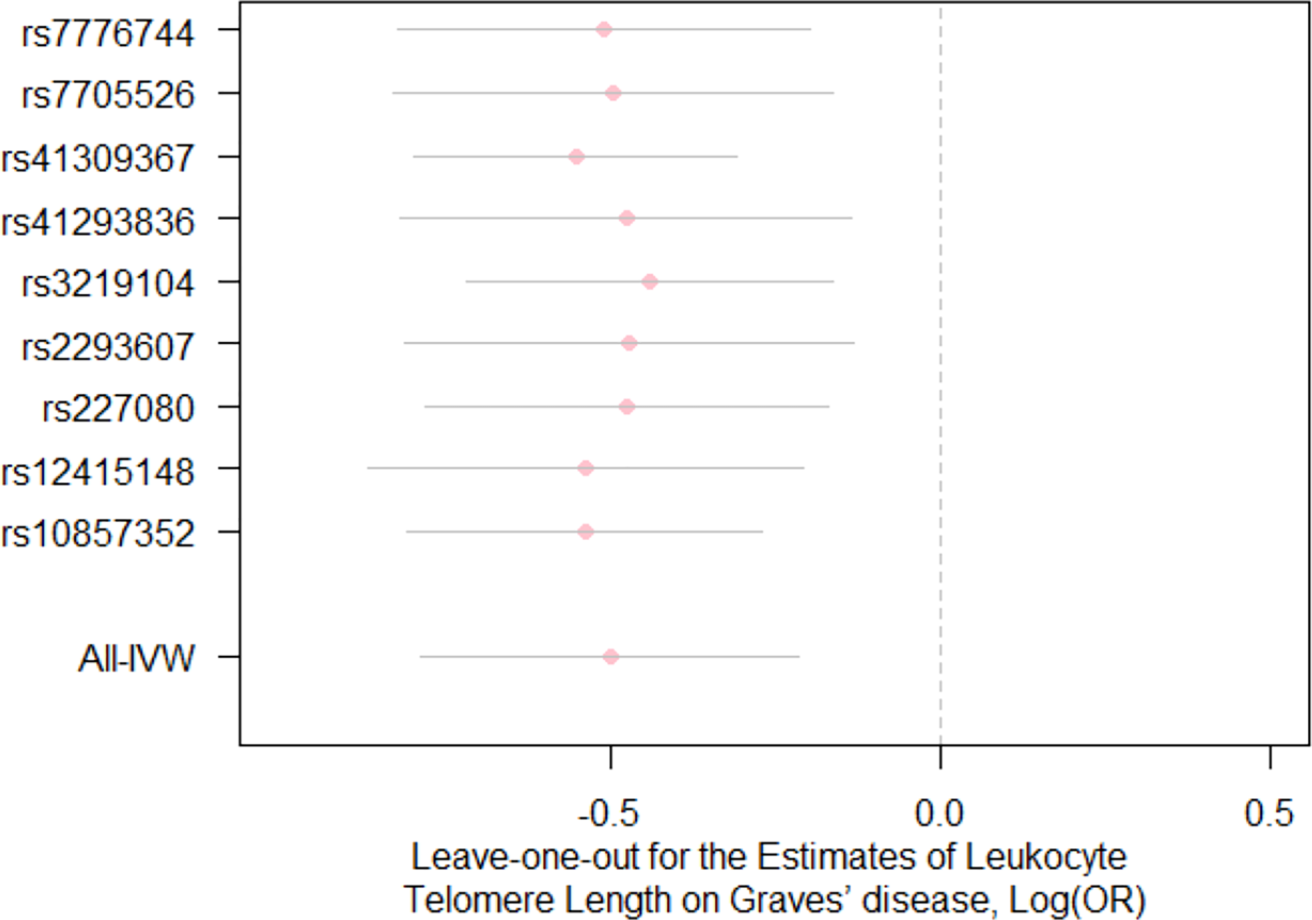
Leave-one-out analysis for the estimates for leukocyte telomere length on the risk of Graves’ disease Each pink dot and grey bar *via* using the IVW method illustrate the effect estimates and 95%CI for telomere length on the risk of Graves’ disease when the indicated SNP was removed. The overall effect estimate and 95% CI are represented by the lowest vertical lines using the IVW method. IVW, inverse variance weighted; OR, odds ratio; CI, confidence interval.

**Figure 5:**
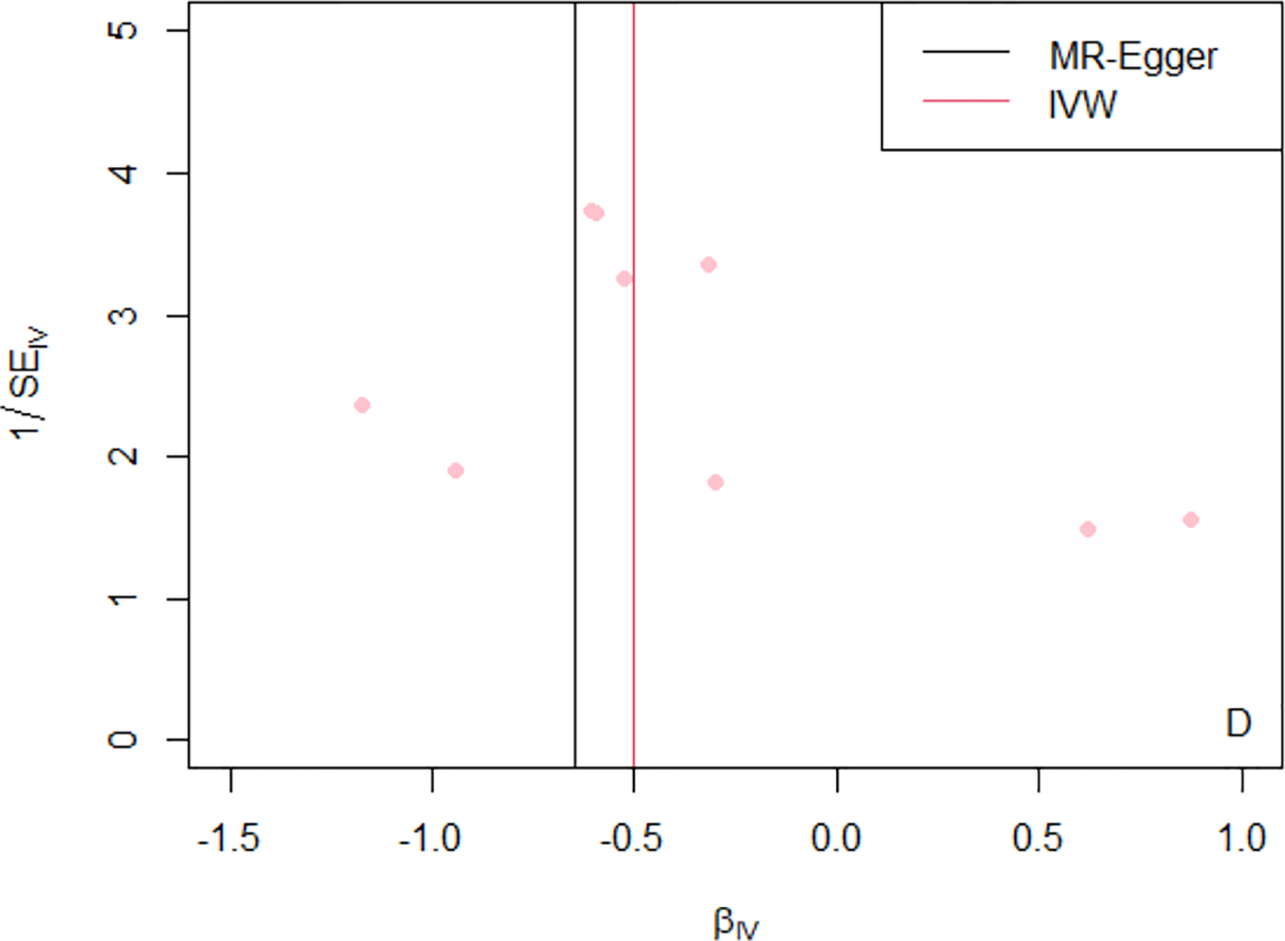
Funnel plot of MR analysis of telomere length on the risk of Graves’ disease by using nine SNPs. The x-axis represents causal estimates (Wald ratios=*β*_Y_/*β*_X_) for each SNP. The y-axis depicts the inverse of standard errors for the effect sizes for each SNP. The overall effect estimates are revealed by vertical lines when using the MR-Egger and IVW approaches (black and pink lines, respectively). SE, standard error; IV, instrumental variable; IVW, inverse variance weighted; MR, Mendelian randomization.

For the heterogeneity test, little evidence of heterogeneity was found by the Cochran’s Q test for IVW and MR-Egger methods (both *P*>0.05). Additionally, for horizontal pleiotropy analysis, the intercept of MR-Egger regression did not provide noticeable evidence (intercept=0.02, se=0.03, *P*=0.641) for horizontal pleiotropy of these SNPs (**Table 2**). The result of MR-PRESSO analysis did not indicate any outliers for any SNPs (*P*-global =0.266). In addition, there was no obvious evidence to support if there was any single SNP that could dominate the results using one SNP plot and leave-one-out analysis (**Figures 3**, **4**). The funnel plot did not provide evidence to support directional pleiotropy from SNPs neither (**Figure 5**).

## Discussion

The present study is the first of this kind to examine the causal association between leukocyte TL and Graves’ disease using a two-sample MR design. By leveraging several MR estimation approaches, we found that shorter TL was associated with an increased risk of Graves’ disease. Our study, in line with previous observational studies, provides evidence to support a causal role of shorter TL for Graves’ disease.

A number of studies about telomere length and lifetime disease risks have been extensively conducted, including autoimmune disorders (35, 36) and age-related diseases (37). Autoimmune diseases are characterized by spontaneous hyperactivity of the immune system comprising autoantibody production. Inflammation and oxidative stress are recognized as parts of the process for most of these diseases, but their molecular mechanisms have not fully been understood, Intriguingly, escalating telomere shorten was primarily caused by environmental factors concerning inflammation and oxidative stress, probably indicating an association between the telomere system and autoimmune or inflammatory diseases (38, 39). Particularly, for the past decade, data emerging from studies in oncology on the telomere system and its underlying therapeutic implications, stimulated studies estimating the role of the telomere system in the biological mechanisms of autoimmune diseases. Indeed, many MR studies utilized telomere length-associated genetic variants to predict the risk of disease based on the genomic architectures of the individuals (40, 41).

To date, no studies, to our knowledge, have been conducted to investigate the role of TL in Graves’ disease. A recent study showed that the relationship between Graves’ disease and rheumatoid arthritis, implying that these two autoimmune diseases were likely to share similar underlying mechanisms (42). Inflammation and oxidative stress are recognized as parts of the process for most of these diseases, but their molecular mechanisms have not fully been understood. Intriguingly, escalating telomere shortening was primarily caused by environmental factors, such as inflammation and oxidative stress, probably indicating an association between the telomeres and autoimmune or inflammatory diseases. Of note, a previous MR study indicated that genetically predicted TL was associated with a lower risk of rheumatoid arthritis, providing support for an inverse causal association (43). A possible biological explanation was that telomerase mutation carriers with short TL may develop T-cell aging phenotypes and immunodeficiency, and T cells with shorter telomeres could induce DNA damage and upregulate intrinsic apoptosis pathways (44). Another explanation was that telomere dysfunction could activate the production and secretion of inflammatory factors, such as IL-6 and TNF-α, which led to cellular senescence (45). Although these explanations are biologically plausible, further studies are required to clarify underlying mechanisms for the role of TL in the risk of Graves’ disease.

Our study has some strengths. First, the MR method tends to be less biased than conventional observational studies in the presence of unmeasured confounding and reverse causation, therefore it can offer a more robust estimate of a causal association. Indeed, this study obtained consistent estimates by using several MR approaches. Second, this study made the best use of publicly available data associated with the GWAS for leukocyte TL (23,096 individuals) and Graves’ disease (2,176 cases and 210,277 controls) with large sample sizes, which rendered us to gain more precise estimates and greater statistical power. Furthermore, the two-sample MR method was taken advantage of both GWAS summary levels with leukocyte TL and Graves’ disease that were derived from two independent populations (46).

Despite the advantages of the MR design, several limitations of the study should be acknowledged. Firstly, the instrumental variables were derived from blood TL and not TL in thyroid tissues in the present study. However, previous studies have shown that leukocyte TL was highly correlated with TL in other tissues (14, 47). Secondly, we cannot prove that the selected SNPs satisfy the assumption of exclusion restriction. However, MR-Egger regression and MR-PRESSO in this study were applied to assess the extent to which genetic pleiotropy may bias the results. The intercept of the MR-Egger regression analysis was close to zero, implying no strong evidence for supporting directional pleiotropy. Similarly, MR-PRESSO and the leave-one-out analysis did not detect significant outliers. Thirdly, it is assumed that the two samples were from the same underlying populations in a two-sample MR study. Lastly, our findings rested on data from GWAS that was only performed in individuals of Asian ancestry, whereas majority of genetic studies were dominated by European-descent samples, which made it difficult to generalize to other ethnic populations. Because of the differences in genetic population structure, the transferability of genetic results across populations is relatively limited. The validity of our analysis relies on the MR assumption that SNPs are not associated with confounders that are related to the occurrence of Graves’ disease. To minimize bias due to violations of this assumption, we reviewed previous articles regarding telomere length and Graves’ disease and screened risk factors related to both as potential confounders, which included smoking status, vitamin D deficiency, and immune status. We found that the instrumental variables were not associated with these potential confounders. Additionally, we screened these instrumental variables through GWAS catalog to try to identify potential genome-wide significant associations with other traits in Asian population, but we did not find any such traits.

## Conclusions

In summary, in the largest study until now, we provided novel evidence to support a causal association of genetically predicted shorter leukocyte TL with an increased risk of Graves’ disease. Further studies are warranted to clarify a biological mechanism of telomeres in the disease onset and progression of Graves’ disease in both the Asian and other ethnic populations.

## Data availability statement

The original contributions presented in the study are included in the article/supplementary material. Further inquiries can be directed to the corresponding author.

## Author contributions

YZ designed this study and collected data, and MY and YW performed the analysis and wrote the manuscript. All authors approved the final version of the manuscript.

## Funding

This work was supported by Sun Yat-Sen University.

The funder had no role in study design; in the collection, analysis and interpretation of data; in the writing of the report; and in the decision to submit the article for publication.

## Conflict of interest

The authors declare that the research was conducted in the absence of any commercial or financial relationships that could be construed as a potential conflict of interest.

## Publisher’s note

All claims expressed in this article are solely those of the authors and do not necessarily represent those of their affiliated organizations, or those of the publisher, the editors and the reviewers. Any product that may be evaluated in this article, or claim that may be made by its manufacturer, is not guaranteed or endorsed by the publisher.
